# Time Investment in Drug Supply Problems by Flemish Community Pharmacies

**DOI:** 10.3389/fphar.2017.00568

**Published:** 2017-08-23

**Authors:** Elfi De Weerdt, Steven Simoens, Minne Casteels, Isabelle Huys

**Affiliations:** Clinical Pharmacology and Pharmacotherapy, Department of Pharmaceutical and Pharmacological Sciences, KU Leuven Leuven, Belgium

**Keywords:** drug shortages, drug supply problems, community pharmacies, time investment, time and motion study

## Abstract

**Introduction:** Drug supply problems are a known problem for pharmacies. Community and hospital pharmacies do everything they can to minimize impact on patients. This study aims to quantify the time spent by Flemish community pharmacies on drug supply problems.

**Materials and Methods:** During 18 weeks, employees of 25 community pharmacies filled in a template with the total time spent on drug supply problems. The template stated all the steps community pharmacies could undertake to manage drug supply problems.

**Results:** Considering the median over the study period, the median time spent on drug supply problems was 25 min per week, with a minimum of 14 min per week and a maximum of 38 min per week. After calculating the median of each pharmacy, large differences were observed between pharmacies: about 25% spent less than 15 min per week and one-fifth spent more than 1 h per week. The steps on which community pharmacists spent most time are: (i) “check missing products from orders,” (ii) “contact wholesaler/manufacturers regarding potential drug shortages,” and (iii) “communicating to patients.” These three steps account for about 50% of the total time spent on drug supply problems during the study period.

**Conclusion:** Community pharmacies spend about half an hour per week on drug supply problems. Although 25 min per week does not seem that much, the time spent is not delineated and community pharmacists are constantly confronted with drug supply problems.

## Introduction

Shortages of “human medicinal products”, later referred to as “drug” shortages, are no longer a new phenomenon (Baumer et al., 2004; Fox et al., 2009; Pharmaceutical Group of the European Union, 2012; Collins, 2013; European Association of Hospital Pharmacists, 2014; De Weerdt et al., 2015a, 2017; Pauwels et al., 2015). Different wordings are used to describe drug supply problems and additional different interpretations are perceived when discussing drug shortages (De Weerdt et al., 2015b). In this paper, the wording “drug supply problem” is used, a term that covers “drug supply disruptions” (i.e., when the supply of drugs does not meet the demand at the level of pharmacies and wholesalers) and “drug shortages” (i.e., when the supply of drugs does not meet the demand at consumer/patient level).

Various causes of drug supply problems are described in literature (European Medicines Agency, 2012; Barlas, 2013; Birgli^®^, 2013; Gupta and Huang, 2013; De Weerdt et al., 2015c). Reputational damage, economic and health implications, increased workload are just a few examples on how drug supply problems affect different stakeholders in the supply chain of delivering the medicinal products to patients (Fox et al., 2009; IMS, 2011; Kaakeh et al., 2011; Ventola, 2011; Havrilesky et al., 2012; Metzger et al., 2012; Pharmaceutical Group of the European Union, 2012; APB, 2015; De Weerdt et al., 2017).

**FIGURE 1:**
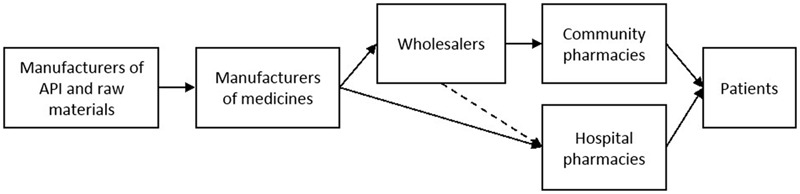
Supply chain of manufacturing and delivering human medicinal products. API = Active Pharmaceutical Ingredient; - - - - - = only a small part of the total products ordered by hospital pharmacies are ordered via wholesalers.

**Figure 1** displays a simplified supply chain for human medicinal products in Belgium. Two types of wholesalers exist in Belgium: (i) general wholesalers and (ii) full-line wholesalers. Full-line wholesalers, in contrast to general wholesalers, have several obligations and rights. For example, full-line wholesalers have the right to be supplied by the pharmaceutical companies. In turn, full-line wholesalers have the obligation to supply community pharmacies and to have a stock of two-third of all medicines available in Belgium (art 101) (Royal Decree Concerning Medicines for Humand and Veterinary Use, 2006).

In Belgium (including Flanders) hospital pharmacies order the majority of their products directly from the manufacturer. Only a small number of products available in hospital pharmacies are ordered by full-line wholesalers. Community pharmacies, on the other hand, must order their products via wholesalers (art 3) (Belgian Law on Medicines, 1964). Most frequently, a community pharmacy co-operates with one main full-line wholesaler and one back-up full-line wholesaler. In Belgium, a distinction is made between private and cooperative community pharmacies. Cooperative pharmacies engage for the purchase and distribution of medicinal products, resulting in mutual benefits for the pharmacies and patients. Due to the cooperation, pharmacies are able to exchange medicinal products between cooperative pharmacies within the same cooperation, which may be very beneficial for patients in case of short-term supply disruptions.

According to previous papers, European community pharmacies spend a lot of time on managing drug supply problems to avoid patients’ impact (Collins, 2013; APB, 2015; De Boer et al., 2015). At European level, a survey concludes that community pharmacists spend between 3 and 15 h a month on drug shortages (Pharmaceutical Group of the European Union, 2012; Medicine Shortages, 2016). Results of a previous questionnaire in the United Kingdom indicate a time spent of less than 1 h to more than 10 h per week on drug supply problems (Collins, 2013). However, it remains unclear which step in the management process consumes most time. Therefore, the aim of this study is to quantify the time employees of Flemish community pharmacies spend on drug supply problems as well as to investigate in detail which steps are most time consuming. In this way, sustainable measures can be formulated to mitigate the problem of drug supply problems.

## Materials and Methods

### Study Design

Time spent by employees of Flemish community pharmacies was studied through a time-and-motion study, which is a non-interventional descriptive study (Zheng et al., 2011). Employees of the community pharmacies were asked to register all the time they spent on managing supply disruptions and drug shortages. Approval for this study was received from the Ethics Committee of the University Hospitals Leuven (S58539).

In Belgium, community pharmacies are obliged to order their medicinal products from wholesalers (art. 3) (Belgian Law on Medicines, 1964). Most community pharmacies have one main full-line wholesaler and one back up full-line wholesaler. If supply problems are experienced for a product, community pharmacies can contact other wholesalers to purchase the particular product or a substitute after consultation with the prescribing healthcare professional (art. 3 and art. 31) (Federale Overheidsdienst Justitie, 1967; Coordinated Law Concerning the Practice of Health Care Professions, 2015). Only when a supply disruption is the result of quota, community pharmacists can contact the manufacturer to purchase the product (art. 12) (Belgian Law on Medicines, 1964). Pharmaceutical industry introduced quota to avoid parallel export. Quota are precisely calculated amounts based on the needs of the Belgian population, including a small margin to ensure continued supply and avoid surpluses to limit parallel export (Sapentis, 2013; De Weerdt et al., 2015c).

Substitution of medicinal products is only allowed when the prescription states the international non-proprietary name (INN) (art. 3 and art. 31) (Federale Overheidsdienst Justitie, 1967; Coordinated Law Concerning the Practice of Health Care Professions, 2015), or if the prescription concerns antibiotics or antimycotics for short-term use. If the Belgian market has no therapeutic alternatives available, community pharmacists can order a substitute abroad. However, this is only possible for prescription medicinal products and if a statement of a healthcare professional is obtained (art. 14) (Royal Decree Regarding Instructions for Pharmacists, 2009). Foreign therapeutic alternative medicinal products are not always considered for reimbursement.

Through a convenience sample of pharmacists in the region of Leuven, three exploratory interviews were conducted to draft a template containing the different steps a community pharmacist and his personnel can undertake to manage drug supply disruptions. The interviews were based on a template from a previous study on time spent on drug supply problems in hospital pharmacies (De Weerdt et al., 2017). Six randomly selected community pharmacies validated the template. Minor improvements were implemented and consensus on this final draft was achieved. An English version of the template is presented in **Table 1**. The template is divided in three sections: (i) time spent on collecting information regarding the supply problem, (ii) time spent on obtaining a substitute, and (iii) time spent on settling the supply problem.

**Table 1:** Template to report time spent on drug supply problems.

	Pharmacist (min)	Pharmacy technicians (min)
Collecting information about drug shortages		
Check missing products from order		
Contact wholesaler or manufacturer regarding possible drug supply problem		
Check stock to see if a drug shortage can cause a problem		
Check if there is a generic or biosimilar available in the pharmacy		
Check if there is an alternative treatment available in the pharmacy		
Contact and consult with prescribing medical doctor		
Pharmaceutical compounding at pharmacy		
Contact wholesaler or manufacturer to place order		
Contact other pharmacies		
Contact international pharmacy to order drugs from abroad		
Set consumer price for drugs from abroad		
Communication with other colleagues		
Communication with patients		
Adjusting medication schedule		
Other: ………………………		

Time spent for drug supply problems was measured. In this study, the wording “drug supply problems” covers “drug supply disruptions” and “drug shortages.” A “supply disruption” was defined as “when the supply of a standard product is interrupted,” meaning the time spent on quota was also included in this study. However, this “supply disruption” does not necessarily lead to a drug shortage in the community pharmacy. A “drug shortage” was defined as a “supply problem which leads to changing the standard therapy into a substituted therapy.” A substituted therapy could be a substitution between generic or biosimilar medicines, changing to an alternative therapy or postpone the therapy if no substitute was available.

Blank templates for daily reporting during the study period were assembled in a map. The template needed to be filled in daily with the total time spent on supply problems, hence not specific per supply problem.

### Sample Size Calculation

To obtain a representative sample size, the Association of Belgian Pharmacists (ABP) provided the numbers of the distribution of private community pharmacies in Flanders. The sample size was calculated considering: (i) the ratio private and cooperative pharmacies in Belgium; (ii) distribution of private community pharmacies in Flanders; (iii) a response rate of 20%; and (iv) the desired number of 35 participating community pharmacies (30 private community pharmacies and five cooperative). Based on these figures, the authors asked ABP to provide a list of 150 randomly selected private community pharmacies. At the beginning of November 2015, an invitation was sent to the 150 private community pharmacies with the request to participate in this study. Due to a low response rate, pharmacists who did not respond within 1 week were contacted in order to reach the desired number of 30 participating private community pharmacies.

Cooperative community pharmacies were gathered through Ophaco (association of cooperative pharmacies in Belgium). The invitation letter was posted on the intranet website of Ophaco. However, since there was no response to the intranet message, cooperative pharmacists were randomly selected and contacted by telephone to ask whether they would like to participate in the study. Eventually five cooperative community pharmacies consented to participate in the study.

### Data Collection

The study started on November 30, 2015 and ended April 3, 2016. Participating pharmacists were contacted for a kick-off meeting before the start of the study for the provision of further information about the study and to deliver the map, containing the templates for all days during the study period as well as templates for 1 week before the study period. This extra week was added in order to familiarize the employees of the pharmacies with the reporting system; if they encountered difficulties, these could be solved before the study period.

Completed templates were collected twice. In February 2016, templates from November 2015 until January 2016 were collected and the remaining templates were collected after the study ended, in April 2016. In March 2016, additional information which might be useful for the analysis of the results was gathered through a questionnaire. This additional information included questions on the location of the pharmacy, the number of patients visiting the pharmacy per day, the number of prescriptions dispensed per day, the stock management and the number of employees. The questionnaires were collected during the last visit. Community pharmacies contributed voluntarily and were not remunerated.

### Data Analysis

The time spent per week on each action to manage drug supply problems was collected in a database (Microsoft Excel 2013). Primary, descriptive analysis was performed due to a relatively small sample size. Medians and interquartile ranges (IQR) were used to express the variables, as the variables were not normally distributed. To control for potential correlation factors, the Pearson correlation was calculated.

Some of the steps as stated in the template (see **Table 1**) were grouped under a common denominator. Under “gathering information” the following steps were considered: (i) check missing products from order; (ii) contact wholesaler or manufacturer regarding possible drug shortage; and (iii) collecting information about drug shortages. Under “search alternative treatment” the following steps were considered: (i) contact and consult with prescribing medical doctor; (ii) check if there is a generic or biosimilar available in the pharmacy; and (iii) check if there is an alternative treatment available in the pharmacy. “Ordering alternative treatment” contains: (i) contact wholesaler or manufacturer to place order; (ii) contact other pharmacies; and (iii) contact international pharmacy to order drugs from abroad. “Communication with patients” and “communication with other colleagues” were combined as “communication toward patients and colleagues.”

Community pharmacies were excluded if they admitted or self-reported not to fill in the templates correctly. Preliminary results were shared with the participating community pharmacies via email and employees of the pharmacy were asked to validate the observed results and trends through a questionnaire.

After a preliminary analysis of the data on the time spent on drug supply problems, community pharmacists were asked to validate the observed results and trends through email.

## Results

A total of 35 community pharmacies started the study (about 1.33% of the Flemish community pharmacies), including 30 private community pharmacies and five cooperative community pharmacies. Eventually, 25 (i.e., 23 private and 2 cooperative community pharmacies) were retained for further analysis, which represents about 0.95% of the Flemish community pharmacies. Six community pharmacies dropped out and four community pharmacies were excluded based upon the exclusion criteria.

The starting sample (*N* = 35) and the final 25 pharmacies are representative to the Flemish situation in terms of the geographical distribution. Concerning the type of pharmacies, an underrepresentation is detected for cooperative community pharmacies. In 7 of the 25 community pharmacies included, only one pharmacist is employed. In 11 community pharmacies, the number of full time equivalent (FTE) pharmacists employed range from 1.25 to 2. Six pharmacies employ three FTE pharmacists and only one participating community pharmacy employ five FTE pharmacists.

**FIGURE 2:**
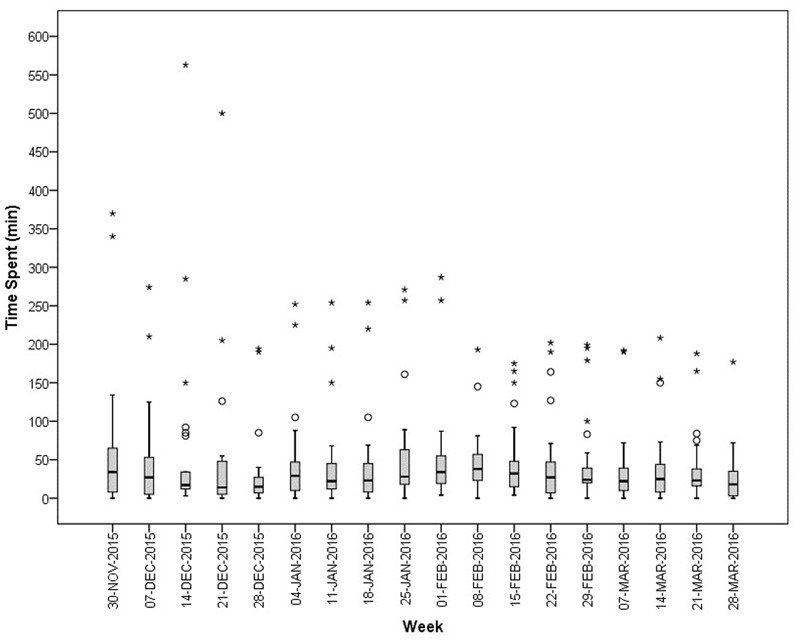
Median (IQR) time spent (in min) per week on supply problems by community pharmacies in Flanders (*N* = 25). ° = outlier [1.5 IQR – 3 IQR]; ^∗^ = extreme (>3 IQR).

**Figure 2** represents the median time spent by the 25 community pharmacies, together with its IQR. The median time spent by the personnel of a community pharmacy on drug supply problems is 25 min per week, with a minimum of 14 min and a maximum of 38 min per week. The majority of the participating community pharmacists confirms our result, about six pharmacists argue that 25 min per week is too low. The minimum is observed in the week of Christmas (December 21, 2015) and community pharmacists attributed this minimum to more closing days during this period. The maximum of 38 min is observed in the second week of February. According to the participants, this can be explained due to an increase of ill persons or by certain drug shortages which required an increased time investment. This increased time investment is detected, among others, in an increase of communication with patients, informing them about the drug supply problem.

**FIGURE 3:**
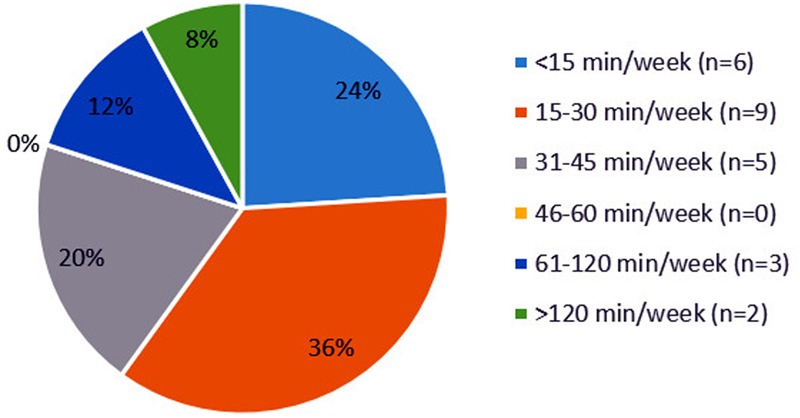
Distribution of community pharmacies (*N* = 25) based on the average time spent on drug supply problems in minutes per week.

In **Figure 2**, several outliers are observed. These outliers represent great differences among the community pharmacies. As displayed in **Figure 3**, enormous differences in the reported times are observed among the 25 community pharmacies. The number of community pharmacies who spend on average less than 15 min per week practically equals the number of community pharmacies who spend more than 60 min per week on drug supply problems. About one-third of the community pharmacies spend on average between 15 and 30 min per week on drug supply problems.

Several aspects of a community pharmacy are considered as potential confounding factors, e.g., the number of patients helped per day, the number of prescriptions dispensed per day, the location of the community pharmacy and the stock management. However, no correlation is found between the total time spent by the community pharmacies and any of these factors (*p* > 0.5).

**FIGURE 4:**
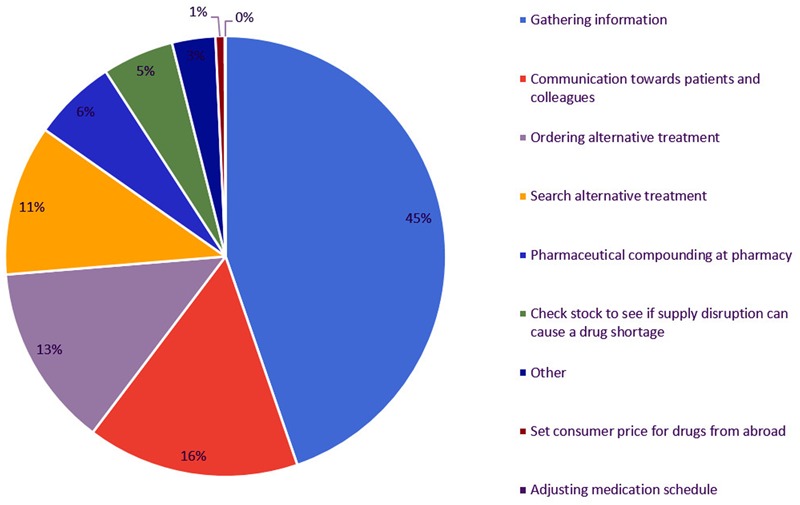
Time spent (% of total time spent) on steps to manage drug supply problems by Flemish community pharmacies.

The most time consuming step is gathering information on drug supply problems, which corresponds to almost half of the total time spent by community pharmacists (**Figure 4**). This step includes checking the orders for missing products, contacting wholesalers and manufacturers and collecting more information on drug shortages via the website of the Federal Agency of Medicines and Health Products (FAMHP). The second most time consuming step is the communication toward patients and colleagues. Patients do not always understand why their product is in shortage and thus why they need to switch to a substitute. Ordering this substitute at another wholesaler or in case of quota at the manufacturer was the third most time consuming step.

Most of the time spent on managing drug supply problems is executed by the pharmacist himself (95%). The remaining 5% of the total time spent is accomplished by the pharmacy technician. It should be noticed that only 11 of the 25 community pharmacies have a pharmacy technician employed. Pharmacy technicians spend most of their time on contacting the wholesaler or manufacturer to receive more information on supply disruptions. Communication toward patients regarding the drug supply problems is the second most time consuming step for pharmacy technicians.

## Discussion

Flemish community pharmacies spend about 25 min on drug supply problems, with a minimum of 14 and a maximum of 38 min per week. The most time consuming step is searching for information on drug supply problems, which corresponds with almost half of the total time spent on managing drug supply problems.

The median time spent on drug supply problems by community pharmacies is much lower than previously reported (Pharmaceutical Group of the European Union, 2012; Collins, 2013). Previous research reveals a range on the time spent by United Kingdom community pharmacists going from less than 1 h a week to more than 10 h per week on managing drug shortages (Collins, 2013). However, of the 371 respondents, only 10% spend less than 1 h on drug shortages. Diverse explanations can clarify the observed difference with our result. First, differences in the applied research methodologies and semantics can result in differences in the time spent on drug shortages. A survey is often more subjective and therefore the result is probably an overestimation, while our study tries to be more objective. Second, national differences in the supply chain of community pharmacies might influence the time spent on drug supply problems.

In the United Kingdom, there are two main supply chains. The first one is similar to the Belgian supply chain (pharmaceutical industry to wholesalers and wholesalers to pharmacies). The second possibility to supply medicines in the United Kingdom is by pharmaceutical industry selling directly to pharmacies (DTP) and paying distributors or wholesalers a fixed fee for delivery (The Association of the British Pharmaceutical Industry [ABPI], 2017). Since the introduction of DTP-model, pharmacists spend apparently a lot more time on sourcing drugs, especially in comparison with the other “standard” supply chain (Kermani, 2010; The Pharmaceutical Journal, 2010). An important strength of the Belgian “standard” supply chain is that full-line wholesalers have medicinal products available of different pharmaceutical manufacturers. The DTP-model complicates the search toward generic or alternative medicines, which might result in an increased amount of time spent by community pharmacies in case of drug supply problems. However, more in-depth research is necessary to investigate the influence of the supply chain on the time spent by pharmacists on drug supply problems.

The time community pharmacists spend on drug supply problems (±25 min per week) also largely differs from the time spent by hospital pharmacists (±2 h per week) (De Weerdt et al., 2017). There are two important factors which can explain this difference: (i) the supply chain of pharmacies (see **Figure 1**) and (ii) the amount of products ordered. In Belgium, most hospital pharmacies order their products directly from the manufacturer and are mostly bound to tender-contracts. The size of the order in combination with limitations due to the contract complicates ordering the product with other manufacturers for hospital pharmacies. Community pharmacies, on the other hand, order their products from wholesalers. Smaller orders and the possibility of easily switching between full-line wholesalers for ordering generic or alternative medicines can clarify the observed difference between the time spent by hospital pharmacies and community pharmacies.

About 90% of the total time spent on drug supply problems is spent by the community pharmacist himself. Taken into account that seven community pharmacies employ only one pharmacist, a median time spent of 25 min per week on drug shortages, can be experienced as a real problem for such pharmacies. Especially since the time spent on drug supply problems is not delineated, but rather a constant reminder of the problem.

The study design has several advantages. By using self-reporting methodology, a relatively high number of community pharmacies could participate in the study (Emmerton and Jefferson, 1996; Rutter et al., 1998). Secondly, our results are based on the time spent by 25 private and cooperative community pharmacies. Thirdly, since drug supply problems vary over time, the widespread study period of 18 weeks is another advantage. However, on the other hand, this study period does not allow observing yearly fluctuations. A fourth bonus of this study consists of the profound way the issue of drug supply problems has been studied, which resulted in suggesting targeted measures (see further) to mitigate the problem.

### Study Limitations

Despite its advantages, this study has also its limitations. Six community pharmacies dropped out of the study because it was too time consuming, four other community pharmacies were excluded based on the exclusion criteria. There is a risk that also other community pharmacies experienced similar problems, but did not admit resulting in an underestimation of the total time spent on drug supply problems.

Participants might have experienced their contribution as troublesome and time-consuming, leading to a risk of underreporting, especially when participants are burdened with work (Emmerton and Jefferson, 1996; Rutter et al., 1998). When validating our results, some participating pharmacists admitted they sometimes forgot to fill in the templates correctly and therefore the median time spent on drug supply problems is an underestimation.

Different interpretation of the wording used in the template should be considered as a third limitation of the study. This can result in inaccurate reporting of the time spent between the different steps. However, the total time spent on drug supply problems by community pharmacists will remain the same.

Our study suffers from a limited sample size of 25 community pharmacies. Since the templates were filled in manually and subsequently transferred manually to an Excel database, the sample size was restricted to guarantee the feasibility of the study. Additionally, the response rate was very low, as explained above, which resulted in originally 35 participating community pharmacies from which 10 dropped out or were excluded.

Although our sample is representative of the population of pharmacies in Belgium, no statistical formulae were applied to calculate the sample size or to test hypotheses given that there were no prior data about the time spent on drug supply problems in community pharmacies in Flanders. The data of our exploratory study may be informative for sample size calculations in future studies.

### Potential Solutions

According to our results, gathering information is the most time consuming step and includes the following steps: (i) checking missing products from order; (ii) contacting wholesaler(s) or manufacturer regarding drug supply problem, and (iii) communication toward patients. Almost half of the total time spent on drug supply problems is spent on finding reliable information about the drug supply problem. By improving the communication between different stakeholders, together with the provision of reliable information about drug supply problems, the total time spent on drug supply problems can be reduced.

Market authorization holders (MAHs) are obliged to report drug shortages to the national competent authorities of the member states of the European Union (Directive 2001/83/EC, art. 23a) (The European Parliament and the Council of the European Union, 2011). To ensure up to date and reliable information of the current supply problems, reporting systems should be user friendly for all parties (MAHs, national competent authorities, etc.). When MAHs fail to report drug shortages, member states should consider introducing sanctions (e.g., financial penalty). In Belgium, a proposal to introduce such penalties when MAHs do not report appropriately is now discussed (Yoleen Van Camp, 2017).

In Belgium, the FAMHP publishes daily a list of the reported drug shortages on their website (Federaal Agentschap voor Geneesmiddelen en Gezondheidsproducten, 2014), but this list contains only drug shortages for which the supply is interrupted for more than 14 days. Drugs subjected to quota are not recorded in this list, however, another website^1^ publishes a list of drugs which experience supply problems due to quota (Koninklijke Apothekersvereniging van Antwerpen [KAVA], 2009). Currently, both lists are not user friendly and are therefore not often consulted by pharmacies. Integrating these lists into the software program of pharmacies would be a great help and could reduce the total time spent by community and hospital pharmacies.

Substitution is not allowed by the Belgian legislation, unless for INN prescriptions. Exceptions are made for prescriptions of antibiotics or antimycotics in an acute treatment, and these prescriptions are always considered as INN prescriptions (Federale Overheidsdienst Justitie, 1967). The legislation on substitution implies that patients with a prescription of a drug experiencing supply problems are informed at the community pharmacy about the shortage. The prescribing physician needs to be contacted by the pharmacist in order to change the prescription, or to be revisited by the patient. Promoting INN prescriptions or allowing substitution in case of shortages are potential solutions to reduce the burden experienced by community pharmacists, prescribing physicians, and patients.

## Conclusion

This study provided exploratory results on the time spent by Flemish community pharmacists on drug supply problems as well as on the most time-consuming tasks within pharmacies. The sample of community pharmacies is relatively small, but representative for the Flemish situation and shows a large variation in the time spent. Though a median of 25 min per week seems not that much, the time spent on drug supply problems is not delineated and community pharmacists are constantly reminded of the problem. Further research in a larger sample of community pharmacies is needed to validate the results of this study. However, improving the provision of information about drug supply problems can reduce the total time spent by pharmacists. Similar results and recommendations were found for Belgian hospital pharmacies (De Weerdt et al., 2017).

## Author Contributions

All authors participated in the design of the study. ED performed data collection and analyzed the data and drafted the manuscript. IH, SS, and MC revised the manuscript critically and contributed to the interpretation of the data. All authors read and approved the final manuscript.

## Conflict of Interest Statement

The work was financially supported by TEVA and the Research Foundation Flanders (FWO). The research was carried out independently by KU Leuven. None of the authors is employed by TEVA. MC is member of the Scientific Advice Working Party at EMA. The other authors declares that the research was conducted in the absence of any commercial or financial relationships that could be construed as a potential conflict of interest.

## Footnotes

1 http://www.farmacontingentering.be/
